# Dietary diversity and social determinants of nutrition among late adolescent girls in rural Pakistan

**DOI:** 10.1111/mcn.13265

**Published:** 2021-08-31

**Authors:** Jo‐Anna B. Baxter, Yaqub Wasan, Muhammad Islam, Simon Cousens, Sajid B. Soofi, Imran Ahmed, Daniel W. Sellen, Zulfiqar A. Bhutta

**Affiliations:** ^1^ Centre for Global Child Health Hospital for Sick Children Toronto Ontario Canada; ^2^ Department of Nutritional Sciences University of Toronto Toronto Ontario Canada; ^3^ Centre of Excellence in Women and Child Health Aga Khan University Karachi Pakistan; ^4^ Department of Infectious Disease Epidemiology London School of Hygiene and Tropical Medicine London UK; ^5^ Dalla Lana School of Public Health University of Toronto Toronto Ontario Canada; ^6^ Department of Anthropology University of Toronto Toronto Ontario Canada

**Keywords:** adolescent girls, diet, food intake, malnutrition, micronutrients, Pakistan, social determinants of health

## Abstract

The conditions in which adolescent girls mature shape their health, development and nutrition. Nutrient requirements increase to support growth during adolescence, but gaps between consumption and requirements exist in low‐ and middle‐income countries. We aimed to identify and quantify the relationship between dietary intake and diverse social determinants of nutrition (SDN) among a subset of adolescent girls 15–18.9 years (*n* = 390) enrolled within the Matiari emPowerment and Preconception Supplementation (MaPPS) Trial. The primary outcome, dietary diversity score (DDS), was derived by applying the Minimum Dietary Diversity for Women 10‐item scale to 24‐h dietary recall data collected three times per participant. To examine the associations between the SDN‐related explanatory variables and DDS, we generated a hierarchical, causal model using mixed effects linear regression to account for the cluster‐randomized trial design. Using all data, diets lacked diversity (DDS mean ± SD: 3.35 ± 1.03 [range: 1–7; *n* = 1170]), and the minimum cut‐off for dietary diversity was infrequently achieved (13.5%; 95% CI: 11.6–15.6%). Consumption of starches was reported in all recalls, but micronutrient‐rich food consumption was less common. Of the SDN considered, wealth quintile had the strongest association with DDS (*P* < 0.0001). The diets of the sampled Pakistani adolescent girls were insufficient to meet micronutrient requirements. Poverty was the most important predictor of a diet lacking in diversity, indicating limited purchasing power or access to nutritious foods. Dietary diversification and nutrition education strategies alone are unlikely to lead to improved diets without steps to tackle this barrier, for example, through fortification of staple foods and provision of supplements.

**Key messages**

- Among the late adolescent girls (15–18.9 years) surveyed in this rural setting in Pakistan, dietary diversity scores were poor, and dietary quality was generally inadequate, suggesting current consumption patterns are unlikely to meet nutrient requirements.
- From the diverse social determinants of nutrition considered, the strongest association was between wealth quintile and dietary diversity score.
- Although diets lacked diversity overall, compared with the poorest participants, those less poor consumed more micronutrient‐rich food groups (e.g., meat and eggs).
- Improving the dietary intake in this setting will require a two‐pronged approach: (1) poverty alleviation strategies are needed to enhance purchasing power, as resource constraints affect many families' ability to access diverse and nutritious food; and (2) micronutrient intake improvement strategies could be of benefit because diet quality is generally poor.

## 1 INTRODUCTION

Adolescent girls' nutrient requirements are increased during puberty to support growth and development, making the availability and consumption of an adequate diet crucial (Patton et al., 2016). Adolescence is also a key life stage for establishing healthy behaviours that can shape both a girl child's current well‐being and that of her adult self and any biological descendants (Black et al., 2013; Viner et al., 2012). Dietary habits track strongly from adolescence into adulthood and can both directly and indirectly affect future offspring (Fatusi & Bello, 2015).

In many low‐ and middle‐income countries (LMICs), particularly in South Asia, adolescent girls' dietary intake and quality are known to be poor, suggesting a gap between consumption and dietary requirements (Aguayo & Paintal, 2017; Keats et al., 2018). Consuming an inadequate diet is an important underlying risk factor for morbidity and mortality (GBD 2017 Diet Collaborators, 2019). Dietary adequacy depends on two key dimensions: diet quantity and quality. Diet quantity reflects an ability to access sufficient energy and macronutrients; diet quality reflects the adequacy of nutrient intake, particularly micronutrients, in meeting biological requirements. Dietary diversity score (DDS) can serve as a proxy for nutrient adequacy, particularly micronutrient sufficiency (Arimond et al., 2010). Quantity, adequacy and diversity of diet are together, or each separately, reduced by any of the many factors that may cause food insecurity.

Within the call for increased understanding of and action on the factors affecting adolescent nutrition, the social determinants of health (SDoH) are highlighted as important drivers of nutritional vulnerability (Christian & Smith, 2018; Fatusi & Bello, 2015). Defined as ‘the conditions in which people are born, grow, live, work, and age’ (Commission on Social Determinants of Health, 2008), SDoH can limit the extent to which someone can access opportunities and resources that affect her health and nutrition. Structural SDoH include the fundamental structures that generate social stratification (e.g., education, ethnicity and wealth), whereas intermediate SDoH include the circumstances within daily life, generated because of structural inequities in access to the benefits of society (e.g., material conditions, biological and behavioural factors, psychosocial circumstances, social exclusion, racism, sexism and stigmatization). Where examined, poverty has been associated with both nutritional status measures and dietary adequacy (Leroy et al., 2018; Venkaiah et al., 2002), and gender‐related inequalities, lack of education, early marriage and empowerment are widely considered to influence adolescent nutrition outcomes (Bhutta et al., 2017; Christian & Smith, 2018). To date, however, investigation of diverse social determinants of nutrition (SDN) among adolescents remains limited, especially in LMICs (Aguayo & Paintal, 2017; Fatusi & Bello, 2015).

In Pakistan, diets are quantitatively inadequate, and micronutrient deficiencies are widespread among married women of reproductive age (WRA; 15–49 years) (UNICEF Pakistan, 2018). From limited data, this is suggested to also be true for adolescent girls (10 to >19 years) (Global Alliance for Improved Nutrition, 2018; Global Alliance for Improved Nutrition & Aga Khan University, 2017). We aimed to identify and quantify the causal relationship between proxy indictors of several key structural and intermediate SDN described in the literature and dietary intake among Pakistani late adolescent girls (15–18.9 years).

## 2 METHODS

### 2.1 Setting and study design

The source data for this analysis are data collected at enrolment into a two‐arm, cluster‐randomized, controlled trial, called the Matiari emPowerment and Preconception Supplementation (MaPPS) Trial, conducted in Matiari District, Pakistan. Matiari district is situated in the north‐eastern part of Sindh province, about 200 km away from Karachi, and representative of typical conditions in rural Pakistan (Baxter et al., 2018a). The Aga Khan University Ethics Review Committee, Research Ethics Board at the Hospital for Sick Children and National Bioethics Committee of Pakistan approved the MaPPS Trial, and it was registered at clinicaltrials.gov.

The MaPPS Trial methodology and data collection tools have been previously described (Baxter et al., 2018b). Briefly, adolescent and young women 15–23 years were identified from a household listing exercise conducted December 2016–May 2017 and later approached at their homes by study personnel to further assess interest and eligibility. Potential participants were eligible if they reported they were not pregnant, able to comply with the intervention, not participating in any other nutrition studies and intend to remain in the study area. Upon confirmation of eligibility, the purpose and voluntary nature of the trial were verbally explained, and an invitation to participate was extended. Written documentation of informed consent was obtained, as well as assent from participants <16 years of age.

Here, we report an analysis of data from late adolescent girls (15–18.9 years) enrolled in a dietary assessment subgroup formed by randomly selecting 390 participants (15 per cluster) < 19 years of age at enrolment, with only one participant per household. Enrolment in the subgroup occurred from July 2017 to April 2018, so as not to include Ramadan when dietary intake and patterns may change drastically.

### 2.2 Conceptual framework

We previously developed a conceptual framework to serve as an overarching guide for understanding how SDN can affect the various components that underlie nutritional status among adolescent girls and the downstream consequences (Appendix S1) (Baxter et al., 2021). The structure was adapted from the UNICEF conceptual framework on the causes of malnutrition and informed by the WHO Commission on SDoH (Commission on Social Determinants of Health, 2008; UNICEF, 1998).

### 2.3 Measures

#### 2.3.1 Dependent variable: DDS

The primary outcome measure was a DDS generated according to the Minimum Dietary Diversity for Women 10‐item scale (Appendix S1) (FAO & FHI 360, 2016). In the 2–3 weeks following enrolment, data on dietary diversity were collected on three separate occasions for each participant using a 24‐h recall method. At each visit, participants were also asked the source of consumed foods over the past week by food group (Appendix S1). Assessment days were unannounced and included two non‐consecutive weekdays and one weekend day. Consumed foods were identified and portion sizes estimated using a food item reference manual and kit (Caulfield et al., 2014). The reference manual allowed for the identification of snacks, branded food items and sizes of whole foods (e.g., mango and roti), whereas the kit included multiple utensils of varying sizes (e.g., spoons, cups and bowls) and a weigh scale. Because covariates were only measured once, an average of the DDS from the three dietary recall repeats was used for each participant in all analyses. To determine adequate intake, also called achieving minimum dietary diversity (MDD), a cut‐off of five or more food groups was applied to participants' mean DDS to generate a binary variable (FAO & FHI 360, 2016). Cumulative measures of DDS and MDD were also generated (Appendix S1).

#### 2.3.2 Explanatory variables: Proxy indicators for SDN

A questionnaire was administered to participants at enrolment to assess diverse SDN‐related explanatory variables hypothesized to affect adolescent nutrition. Information collected included demographics; socio‐economic status; reproductive health and history; nutrition‐related practices; and life skills, empowerment and mental health‐related factors. Questions were adapted from the existing national survey administered in Pakistan and several standardized assessment tools (Appendix S1).

#### 2.3.3 Nutritional status indicators

Nutritional status was assessed from anthropometric measures (height, weight and middle‐upper arm circumference) and a 5‐mL venous blood sample. Standard WHO cut‐offs were applied to determine measures of under‐ and overnutrition (underweight, overweight, obese and stunting) and selected deficiencies of public health concern (anaemia, iron deficiency, iron deficiency anaemia, vitamin A deficiency and vitamin D deficiency; Appendix S1).

### 2.4 Statistical analysis

All enrolment data were collected prior to the start of the study intervention and are analysed as cross‐sectional data. Descriptive statistics for the study population included means with standard deviations (SDs) and counts with proportions for continuous and categorical variables, respectively. A variable for season (summer, rainy or winter) was also generated. Data on the consumption of the 10 food groups were also summarized.

Guided by the a priori conceptual framework, we aimed to generate a hierarchical, causal model to examine the associations between the explanatory variables and average DDS. We hypothesized that there were four levels of SDN explanatory variables: (1) socio‐economic status: education, occupation, religion and wealth quintile; (2) household and personal factors: household food security, marital status and parity; (3) health and well‐being: perception of own health, body image, experience of depression‐, anxiety‐ and stress‐like feelings; and (4) actions and practices: self‐efficacy, participation in decision‐making, skipping breakfast and eating dinner with family. For all analyses, the reference for each variable was set as the category at which someone would be likely to experience the greatest nutritional vulnerability (Baxter et al., 2021).

Crude analyses were first conducted using linear regression for each explanatory variable with average DDS as the dependent variable. Those variables for which the *P* value was <0.20 in the unadjusted analysis were considered potentially relevant for inclusion in the hierarchical model (Hosmer & Lemeshow, 2000). Using an adapted hierarchical model building approach with four levels, relevant variables were entered into a regression model, starting with those at the most distal level (Victora et al., 1997). At each level of the hierarchy, all variables identified for possible inclusion were serially removed using a backward stepwise model building approach to observe any change in the effect estimates and find a model that best explained the variability in the data. For each level, when the *P* value of an explanatory variable in the model was <0.05, it was retained. If the *P* value was ≥0.05 or the standard error (SE) was large, exclusion of the variable from the model was considered based on relative effect size. Specifically, if exclusion of the variable changed the *β* coefficient of the other variables that remained in the model by >10%, the excluded variable was returned to the model. Otherwise, such variables were dropped. The effect estimates presented for retained variables correspond to the point of addition to the model. All analyses were performed using Stata version 15.0. The hierarchical model was fitted using the *mixed* command, to account for the cluster‐randomized design of the MaPPS Trial (26 clusters).

## 3 RESULTS

### 3.1 Participant characteristics

Upon enrolment, the mean (±SD) age of the 390 late adolescent girls enrolled in the MaPPS Trial dietary assessment subgroup was 17.2 ± 1.2 years. Nearly half received no formal education, and 44 (11.3%) were married, with a mean age at marriage of 16.0 ± 1.4 years (Table 1). The primary source of income among participants' households was agriculture or manual labour (83.8%), and 21.0% reported owning agricultural land. Livestock ownership was reported by 57.2% of participants. Nearly all households had electricity (96.2%), and 67.7% owned a television, 35.6% a refrigerator and 6.4% a computer.

**Table 1:** Associations between possible social determinants of nutrition and average dietary diversity score among adolescents enrolled in the MaPPS Trial dietary subgroup (*n* = 390)

Possible determinants of dietary diversity score	*n* (%)	Dietary diversity score	Crude analysis	
		Mean (95% CI)	*β* coefficient (95% CI)	*P* value
Level 1: structural factors—socio‐economic status				
Highest level of education				0.13
None	173 (44.4)	3.28 (3.17–3.38)	‐	
Primary	95 (24.4)	3.34 (3.20–3.49)	0.06 (−0.13 to 0.26)	
Secondary or higher	122 (31.3)	3.45 (3.30–3.62)	0.18 (0.003 to 0.36)	
Occupation				0.005
Unskilled manual labour	85 (21.8)	3.29 (3.13–3.44)	‐	
Skilled manual labour	77 (19.7)	3.12 (2.95–3.28)	−0.17 (−0.41 to 0.07)	
Within the home	161 (41.3)	3.41 (3.29–3.53)	0.13 (−0.08 to 0.33)	
Other	67 (17.2)	3.54 (3.35–3.74)	0.26 (0.01–0.50)	
Religion				0.32
Hindu	32 (8.2)	3.22 (2.98–3.46)	‐	
Muslim	358 (91.8)	3.36 (3.28–3.44)	0.14 (−0.14 to 0.42)	
Wealth quintile				0.0001
Q1 (poorest)	58 (14.9)	2.93 (2.77–3.08)	‐	
Q2	85 (21.8)	3.34 (3.16–3.51)	0.41 (0.16–0.66)	
Q3	93 (23.8)	3.37 (3.21–3.52)	0.44 (0.19–0.69)	
Q4	90 (23.1)	3.39 (3.24–3.54)	0.46 (0.21–0.71)	
Q5 (least poor)	64 (16.4)	3.67 (3.45–3.89)	0.74 (0.47–1.01)	
Level 2: intermediate factors—household and personal characteristics				
Marital status				0.95
Married	44 (11.3)	3.36 (3.16–3.55)	‐	
Unmarried	346 (88.7)	3.35 (3.26–3.43)	−0.01 (−0.25 to 0.24)	
Ever been pregnant				0.44
Yes	21 (5.4)	3.48 (3.19–3.76)	‐	
No	369 (94.6)	3.34 (3.26–3.42)	−0.13 (−0.48 to 0.21)	
Household food security status				0.05
Food insecure	122 (31.3)	3.23 (3.11–3.36)	‐	
Food secure	268 (68.7)	3.40 (3.30–3.50)	0.17 (0–0.33)	
Level 3: intermediate factors—health and well‐being characteristics				
Perception of own health				0.64
Poor or fair	40 (10.3)	3.37 (3.11–3.63)	‐	
Good	244 (62.6)	3.32 (3.22–3.42)	−0.05 (−0.31 to 0.22)	
Excellent	106 (27.2)	3.41 (3.26–3.55)	0.04 (−0.24 to 0.32)	
Body image				0.36
Underweight	69 (17.7)	3.45 (3.26–3.65)	‐	
Normal	289 (74.1)	3.32 (3.23–3.40)	−0.14 (−0.34 to 0.07)	
Overweight	32 (8.2)	3.42 (3.12–3.71)	−0.04 (−0.36 to 0.29)	
Experience of depression‐like feelings				0.09
Severe or extremely severe	20 (5.1)	2.97 (2.69–3.24)	‐	
Moderate	48 (12.3)	3.48 (3.23–3.72)	0.51 (0.11–0.92)	
Mild	30 (7.7)	3.29 (3.00–3.58)	0.32 (−0.12 to 0.76)	
None	292 (74.9)	3.36 (3.27–3.45)	0.39 (0.04–0.74)	
Experience of anxiety‐like feelings				0.20
Severe or extremely severe	49 (12.6)	3.20 (3.01–3.39)	‐	
Moderate	77 (19.7)	3.29 (3.11–3.47)	0.09 (−0.19 to 0.37)	
Mild	26 (6.7)	3.23 (2.89–3.57)	0.03 (−0.34 to 0.40)	
None	238 (61.0)	3.41 (3.31–3.51)	0.22 (−0.02 to 0.45)	
Experience of stress‐like feelings				0.14
Severe or extremely severe	12 (3.1)	3.08 (2.69–3.48)	‐	
Moderate	17 (4.4)	3.20 (2.86–3.53)	0.11 (−0.46 to 0.69)	
Mild	26 (6.7)	3.10 (2.84–3.36)	0.02 (−0.51 to 0.55)	
None	335 (85.9)	3.39 (3.30–3.47)	0.30 (−0.15 to 0.75)	
Level 4: intermediate factors—actions and practices‐related characteristics				
Self‐efficacy				0.70
Low	124 (31.8)	3.31 (3.17–3.45)	‐	
Moderate	196 (50.3)	3.35 (3.24–3.46)	0.04 (−0.14 to 0.21)	
High	70 (17.9)	3.41 (3.21–3.61)	0.10 (−0.13 to 0.33)	
Decision‐making autonomy				0.04
All decisions made by family	234 (60.0)	3.27 (3.17–3.36)	‐	
Most decisions made by family	100 (25.6)	3.49 (3.34–3.65)	0.22 (0.04–0.40)	
Decisions made jointly with family or autonomously	56 (14.4)	3.42 (3.19–3.65)	0.15 (−0.07 to 0.37)	
Skipping breakfast				0.45
Skips breakfast	114 (29.2)	3.39 (3.27–3.52)	‐	
Eats breakfast	276 (70.8)	3.33 (3.23–3.43)	−0.07 (−0.24 to 0.11)	
Eating dinner with family				0.04
Never	87 (22.3)	3.16 (3.01–3.32)	‐	
Sometimes	33 (8.5)	3.44 (3.16–3.73)	0.28 (−0.03 to 0.59)	
Every day	270 (69.2)	3.40 (3.30–3.49)	0.23 (0.04–0.42)	

Abbreviations: CI, confidence interval; MaPPS, Matiari emPowerment and Preconception Supplementation.

Some form of food insecurity was reported at the household level by 31.3% of participants (Table 1). Most of the adolescent girls reported eating dinner with their family at some point during the preceding week; one‐third reported skipping breakfast regularly. A majority were found to have low or moderate self‐efficacy (82.1%), and decision‐making autonomy was limited (1.3%). Most adolescent girls did not report having elevated depression‐, anxiety‐ or stress‐like feelings over the past week (74.9%, 61.0%, and 85.9%, respectively). A majority reported being in good or excellent health (89.8%) and feeling that their weight was ‘normal’ (74.1%).

### 3.2 Nutritional status

Stunting was common (29.7%) (Table 2). Anthropometric measures of body proportions and adiposity indicated most participants were normal or thin (91%). The prevalence of micronutrient deficiencies was high: 43.8% were anaemic, and 35.4% had iron deficiency anaemia. Vitamin A and D deficiency was experienced by 29.6% and 81.1%, respectively. Nearly half (45.7%) of this subsample of participants experienced ≥2 deficiencies of public health concern (iron deficiency anaemia, vitamin A and/or vitamin D deficiency).

**Table 2:** Nutritional status‐related characteristics among participants at enrolment in the MaPPS Trial dietary assessment subgroup (*n* = 390)

Characteristic	Value^a^
Anthropometric measures	
Weight (kg)	46.0 ± 8.6
Height (cm)	152.6 ± 5.9
Height <145 cm	31 (8.0)
Height‐for‐age *z*‐score	−1.5 ± 0.9
Height‐for‐age *z*‐score <−2 SD	116 (29.7)
BMI (kg/m^2^)	19.7 ± 3.3
BMI categorization	
Underweight (<18.5 kg/m^2^)	160 (41.0)
Normal (18.5–24.9 kg/m^2^)	202 (51.8)
Overweight (25–29.9 kg/m^2^)	23 (5.9)
Obese (≥30 kg/m^2^)	5 (1.3)
BMI‐for‐age *z*‐score	−0.6 ± 1.2
BMI‐for‐age *z*‐score categories	
Thin (<−2 SD)	47 (12.1)
Normal (−1 to 1 SD)	311 (79.7)
Overweight (>1 to 2 SD)	26 (6.7)
Obese (>2 SD)	6 (1.5)
Middle‐upper arm circumference (cm)	23.5 ± 2.9
Micronutrient measures	
Haemoglobin concentration (g/dL)	11.9 ± 1.8
Anaemic^b^	171 (43.8)
Serum ferritin concentration (μg/L)	10.6 ± 2.8
Iron deficiency^c^	242 (62.1)
Iron deficiency anaemia^d^	138 (35.4)
Serum retinol concentration^e^ (μmol/L)	0.94 ± 0.44
Vitamin A deficiency^f^	115 (29.6)
Serum 25(OH)D concentration^g^ (ng/mL)	15.1 ± 8.5
Vitamin D deficiency^h^	314 (81.1)
CRP concentration (mg/dL)	0.13 ± 0.26
Acute inflammation^i^	16 (4.1)

Abbreviations: 25(OH)D, 25‐hydroxy‐vitamin D; BMI, body mass index; CRP, C‐reactive protein; MaPPS, Matiari emPowerment and Preconception Supplementation; SD, standard deviation.

^a^ Data are mean ± SD or *n* (%).

^b^ Anaemia defined as haemoglobin <12.0 g/dL.

^c^ Iron deficiency defined as serum ferritin <15 μg/L without acute inflammation (CRP < 5 mg/L) or serum ferritin <70 μg/L with acute inflammation (CRP > 5 mg/L).

^d^ Iron deficiency anaemia defined as iron deficiency and anaemia (haemoglobin <12 g/dL).

^e^ Serum retinol data was not obtained for 2 participants due to insufficient sample (*n* = 288).

^f^ Vitamin A deficiency defined as a retinol concentration <0.7 μmol/L.

^g^ Serum 25(OH)D was not obtained for three participants due to insufficient sample (*n* = 287).

^h^ Vitamin D deficiency was defined as 25(OH)D concentration <20 ng/mL.

^i^ Acute inflammation was defined as CRP > 5 mg/L.

### 3.3 Dietary intake characteristics

Using the pooled dietary recall data on all participants, the mean DDS was 3.35 ± 1.03 (range: 1–7; *n* = 1170), and MDD was achieved 13.5% (95% CI: 11.6–15.6%) of the time. MDD was not achieved for any of the three recalls by 72.1% of participants (Table 3). Starchy staple foods were consumed universally by participants, whereas the consumption of the other food groups varied when pooled. The major food groups consumed included other vegetables (64.8%), other fruits (45.9%) and dairy products (38.9%). Additionally, 90.2% reported consuming sugar‐sweetened beverages (e.g., tea with added sugar and soda), 43.1% sweets (e.g., candy and dessert) and 22.5% savoury or fried snacks (e.g., samosa and pakora). Nearly all participants reported consuming tea daily (93.1%, 1.7 ± 0.7 cups/day). Half of participants (52.3%) consumed areca nuts in at least one of the recalls and 20.0% in all of the recalls. A majority of participants' food was purchased; dairy products were the exception (Table 4). Participants' mean DDS was 3.35 ± 0.78 (range: 1.33–6.0; *n* = 390) and 3.26 ± 1.02, 3.36 ± 1.02 and 3.43 ± 1.06 for each sequential recall (Table S1). When considering someone's intake from all three recalls cumulatively, mean DDS was 4.9 ± 1.2 (range: 2–8), and 63.9% of participants achieved MDD. DDS was similar between the summer, rainy and winter seasons (3.25 ± 0.80, 3.45 ± 0.74 and 3.38 ± 0.77, respectively).

**Table 3:** Dietary intake characteristics determined from all adolescent girls' dietary recall data (*n* = 1170), reflecting consumption on the day prior to the survey^a^

Characteristic	Average	Total number of recalls reported by participants			
		0	1	2	3
Achieved minimum dietary diversity	13.5	281 (72.1)	69 (17.7)	31 (7.9)	9 (2.3)
Food groups consumed					
Starchy staples	100	0	0	2 (0.5)	388 (99.5)
Other vegetables	64.8	31 (7.9)	87 (22.3)	145 (37.2)	127 (32.6)
Other fruits	45.9	148 (37.9)	56 (14.4)	77 (19.7)	109 (27.9)
Dairy	38.9	163 (41.8)	84 (21.5)	58 (14.9)	85 (21.8)
Pulses	32.1	140 (35.9)	148 (37.9)	78 (20.0)	24 (6.2)
Flesh foods	26.1	188 (48.2)	115 (29.5)	71 (18.2)	16 (4.1)
Eggs	10.5	302 (77.4)	61 (15.6)	19 (4.9)	8 (2.1)
Dark green leafy vegetables	9.0	304 (77.9)	69 (17.7)	15 (3.8)	2 (0.5)
Vitamin A rich fruits and vegetables	3.1	378 (96.9)	0	0	12 (3.1)
Nuts and seeds	2.4	369 (94.6)	15 (3.8)	5 (1.3)	1 (0.3)

^a^ Data are % or *n* (%).

**Table 4:** Reported sources of 14 food groups within the DDS, as reported by late adolescent girls (*n* = 390) in the MaPPS Trial dietary assessment subgroup using data from all three food source assessments

Food group	*n* ^a^	Own production	Purchased	Borrowed/credit	Bartered/traded	Worked for food	Gift	Food aid	Other
Foods made from grains	1170	198 (16.9)	905 (77.4)	31 (2.6)	0	32 (2.7)	0	3 (0.3)	1 (0.1)
White roots and tubers	1167	1 (0.1)	1,152 (98.7)	8 (0.7)	2 (0.2)	3 (0.3)	0	1 (0.1)	0
Pulses	1006	1 (0.1)	990 (98.4)	12 (1.2)	0	1 (0.1)	0	2 (0.2)	0
Nuts and seeds	164	0	161 (98.2)	0	0	0	3 (1.8)	0	0
Dark green leafy vegetables	494	18 (3.6)	424 (85.8)	1 (0.2)	2 (0.4)	4 (0.8)	25 (5.1)	2 (0.4)	18 (3.6)
Vitamin A rich vegetables	1146	3 (0.3)	1,134 (99.0)	6 (0.5)	2 (0.2)	0	0	1 (0.1)	0
Vitamin A rich fruits	301	4 (1.3)	294 (97.7)	0	0	0	3 (1.0)	0	0
Other vegetables	863	4 (0.5)	848 (98.3)	7 (0.8)	0	1 (0.1)	0	0	3 (0.3)
Other fruits	434	3 (0.7)	417 (96.1)	0	0	4 (0.9)	7 (1.6)	2 (0.5)	1 (0.2)
Meats and poultry	826	19 (2.3)	766 (92.7)	0	0	2 (0.2)	21 (2.5)	18 (2.2)	0
Organ meats	104	11 (10.6)	84 (80.8)	0	0	0	5 (4.8)	4 (3.9)	0
Eggs	507	17 (3.4)	487 (96.1)	3 (0.6)	0	0	0	0	0
Fish and seafood	312	1 (0.3)	311 (99.7)	0	0	0	0	0	0
Milk and milk products	760	309 (40.7)	441 (58.0)	3 (0.4)	0	1 (0.1)	3 (0.4)	3 (0.4)	0

Abbreviations: DDS, dietary diversity score; MaPPS, Matiari emPowerment and Preconception Supplementation.

^a^ *N* corresponds to the sum of the participants who reported consuming the food group in the past week from all possible assessment data (maximum is 1170). No significant difference in the reported sources of food was found between the three assessments for each food group, with the exception of nuts and seeds (recall 1: 100% reported purchase; recall 2: 100% reported purchase; recall 3: 94.1% reported purchase and 5.9% reported gift; *P* = 0.03).

### 3.4 Crude analyses

Among the socio‐economic variables considered within Level 1 of the hierarchical model, there was evidence that occupation and wealth quintile were associated with DDS in the unadjusted analyses (Table 1; *P* = 0.005 and *P* < 0.0001, respectively). There was evidence that living in a food secure household was associated with higher DDS (*P* = 0.05) in the unadjusted analysis from the variables considered for Level 2. Among the health and well‐being variables included within Level 3, none were associated with DDS. For the Level 4 variables related to actions and practices, there was some evidence that higher DDS was associated with contributing to decision‐making and eating dinner with family in the preceding week in the unadjusted analysis (*P* = 0.02 and *P* = 0.04, respectively).

### 3.5 Generating the causal hierarchical model

After fitting the hierarchical model, starting with the most distal level, one variable (wealth quintile) was maintained in the final model (*P* value for model: <0.0001). At Level 1, education, occupation and wealth quintile qualified for inclusion from the univariate analyses; however, when included in the multivariate model, the *P* values for education and occupation were 0.47 and 0.89, respectively, and their SEs were large. Their stepwise removal did not affect the *β* coefficient of remaining variables (i.e., wealth quintile), and they were dropped. Household food security qualified for inclusion from Level 2, although upon inclusion, its *P* value was 0.70, and the SE was large. Its stepwise removal also led to its exclusion, as it did not affect other variables considered in the model. For Level 3, experiencing depression‐, anxiety‐ and stress‐like emotions were included in the model. In the multivariate model, their *P* values were 0.88, 0.65 and 0.46, respectively, and their stepwise removal did not affect estimates. For Level 4, the univariate associations with DDS for decision‐making autonomy and eating dinner with family led to their inclusion; yet, in the multivariate model, their *P* values were 0.65 and 0.57, respectively, and they were serially removed. Adjustment for the season of data collection did not affect the effect size of the variables considered in the model, and there was no evidence of an interaction between season and wealth quintile (*P* = 0.42).

### 3.6 Inequalities in dietary consumption

**Figure 1:**
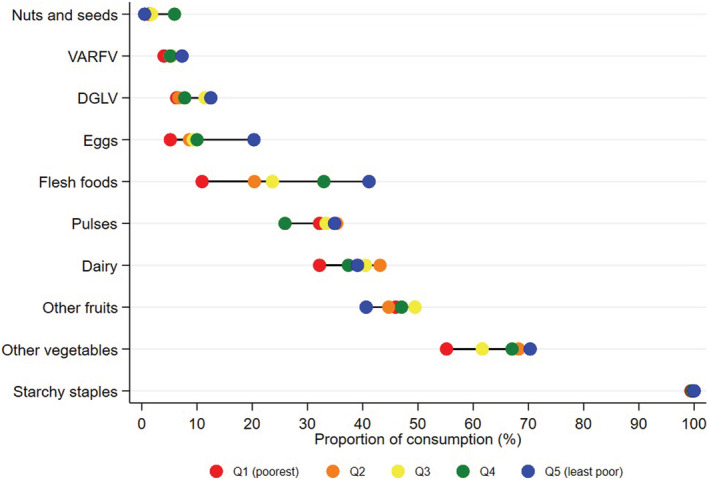
Reported consumption of the 10 food groups, disaggregated by wealth quintile, using all dietary recall data (*n* = 1170). Abbreviations: DGLV, dark green leafy vegetables; flesh foods, meat, poultry and fish; starchy staples, grains, white roots and tubers; VARFV, vitamin A‐rich fruits and vegetables

Being from the least poor quintile had the strongest association with increased DDS. The proportion of participants who reported consuming the 10 food groups generally differed most between the poorest and least poor wealth quintiles (Figure 1). There was overlap between wealth quintiles for the food groups for vitamin A rich fruits and vegetables, dark green leafy vegetables and other fruits. Disparities were notable for the consumption of eggs and other vegetables (e.g., tomatoes, onion and pepper) and widest for the reported consumption of flesh foods (poorest: 10.9% vs. least poor: 41.1%). This gradient was maintained when food group consumption data were considered cumulatively (Figure S1).

## 4 DISCUSSION

Among late adolescent girls living in Matiari District, in rural Pakistan, we observed that the majority consumed monotonous diets and did not meet the cut‐off for MDD. A hierarchical, causal model of SDN and DDS indicated relative poverty, assessed using an asset quintile and had the strongest association with poor diet. DDS was also low among those in the least poor wealth quintile. To our knowledge, this is the first study to investigate the relationship between diverse SDN, at both the structural and intermediate levels, and dietary intake among adolescent girls in a LMIC setting. We believe it is plausible that the observed association between relative poverty and DDS reflects a causal relationship. Findings demonstrate the importance of widespread underlying poverty in accessing nutritious food.

Our results are underpinned by rigorous dietary assessment methods account for seasonality and include the largest population‐based cohort of Pakistani adolescent girls to date. When compared with national estimates, estimates for WRA in rural Sindh province, DDS was comparable (3.1 vs. 3.35), and MDD was infrequently achieved (11.7 vs. 13.5%). National data on the burden of underweight were similar (11.4 vs. 12.1%), although fewer study participants experienced anaemia (56.6 vs. 43.8%) (Government of Pakistan, 2020a; UNICEF Pakistan, 2018). Similar to reviews of food intake among Pakistani adolescents, diets were poor (Global Alliance for Improved Nutrition, 2018; Global Alliance for Improved Nutrition & Aga Khan University, 2017), but we further showed they were insufficient to meet micronutrient requirements. We observed that starchy staple foods were universally consumed, but micronutrient‐rich foods were consumed infrequently and varied between wealth quintiles. This was particularly evidenced by the increased consumption of meat and eggs in the least poor quintile, which are nutrient‐dense foods with a high cost. Consistent with large reviews of adolescent dietary habits in LMICs (Beal et al., 2019; Keats et al., 2018), we found that our adolescent participants commonly consumed highly sweetened tea, desserts and fried snack foods.

Although we found evidence of an association between wealth quintile and DDS, DDS appeared similar across the middle quintiles. Importantly, the average DDS for each wealth quintile was lower than the cut‐off for achieving MDD (≥5 food groups), meaning most participants are unlikely to achieve micronutrient sufficiency from available diets alone. Of note, the study area is situated in a fertile area where there is abundant production of diverse fruits and vegetables. However, this did not translate into frequent consumption of these items, and households mainly reported purchasing foods. This is likely because of the predominance of sharecropping, with excess produce being sent to market.

In the full model, wealth became the dominant driver of dietary adequacy. Notably, wealth quintile representation was not equal among participants, as this variable was generated at the level of the household using all data from the larger MaPPS Trial (>17,000 households). The importance of wealth to diet among adolescents and WRA in LMICs has been previously described (Harris‐Fry et al., 2015; Leroy et al., 2018; Nguyen et al., 2014). When considering the undernutrition of WRA and children more broadly, socio‐economic inequalities are known to be contributing factors, and there is stated a role for multisectoral actions to accelerate progress (Victora et al., 2021).

From key informant interviews with Pakistani teachers and health service providers across provinces, Akhtar et al. (2014) identified poverty as a key barrier to nutrition for adolescent girls, as well as their limited nutrition knowledge. Awareness of which foods are important to meet nutritional needs is similarly limited among WRA in Pakistan (Aga Khan University, 2013). There is a need for the government to address the cost of food and enable access to nutritious foods, especially given food‐price inflation (Akhtar et al., 2014). In the pre‐COVID era, the Pakistan Cost of Diet study estimated that two out of three households were unable to afford a nutritionally adequate diet (Government of Pakistan, UK Aid, & UNICEF Pakistan, 2018). There could be a secondary role for promoting dietary modification to improve the consumption of affordable, locally available nutritious foods and improving nutrition knowledge (Akhtar et al., 2014; Global Alliance for Improved Nutrition, 2018; Government of Pakistan, UK Aid,, & UNICEF Pakistan, 2018).

With respect to other structural SDN variables, in the crude analysis, the evidence of an association between participants' education and DDS was weak, although there was a small gradient in DDS as education level increased. Studies in other settings have typically found a positive association between educational attainment and dietary intake (Nguyen et al., 2014; Sinharoy et al., 2018; Sinharoy et al., 2019). However, these studies surveyed married women at a later life stage than the late adolescent girls in our study.

Several intermediate SDN were proposed to influenced DDS within our conceptual framework, including material circumstances (food security) and empowerment (marital status, self‐efficacy and decision‐making participation). We envisioned household food security as a critical component of someone's food environment, in underlying health, actions and practices. DDS was lower among those in food insecure households, although the association was not maintained after accounting for wealth quintile. Among WRA in Bangladesh, Sinharoy et al. (2019) described a relationship between household food security and higher DDS. Within the context of this rural Pakistani population, we did not find a relationship between empowerment and DDS after adjustment. We were unsure what effect being married might have among adolescent participants (e.g., create social stigma with peers and affect status within one's family), although it did not appear to be associated with DDS. Self‐efficacy was not found to be associated with DDS, and the crude association of decision‐making autonomy with DDS was not maintained. Culturally, adolescent girls in rural Pakistan have limited mobility and decision‐making flexibility, which likely affects their voice and agency as it pertains to food choices (Akhtar et al., 2014). Although female empowerment is hypothesized to facilitate a higher DDS in LMICs, given the increased potential for voice and agency among those empowered, elements of female empowerment have shown limited improvements in dietary intake (Leroy et al., 2018; Sinharoy et al., 2018; Sinharoy et al., 2019; Sraboni & Quisumbing, 2018). In other societies and contexts where adolescent girls experience more autonomy, certain SDN may be important to dietary intake given the different distribution of risk factors within populations. Nonetheless, in the face of poverty, empowerment may have a restricted effect on reducing micronutrient deficiencies, highlighting the importance of resource constraints as a limiting factor.

Although we did not find evidence that nutritional vulnerability was associated with most of the diverse SDN proposed, this may not reflect all contexts and settings. The SDN assessments required self‐reporting, and we cannot rule out measurement error in assessing exposures, such as social desirability bias. Alternatively, it is possible we did not assess all relevant SDN for this context. We aimed to prioritize factors previously identified as related to nutrition outcomes and did not include macroeconomic and policy contexts, although these also underlie SDN. The primary outcome measure (DDS) may also have been subject to recall bias, as it was derived from 24‐h recall data. Given the repetitiveness of diets in the study area and the use of a multiple pass approach, including memory cues to enhance complete and accurate food recall, this would ideally have been minimized. Although validated in multiple settings for assessing micronutrient intake, DDS does not represent caloric intake, nor the macronutrient quality of foods (Arimond et al., 2010).

Addressing poverty and undernutrition are essential components of the Sustainable Development Goals and enabling the consumption of healthy and sustainable diets will be critical to achieving goal two (zero hunger) (United Nations, 2015). We believe further investigation of the food environment (e.g., accessibility and affordability) will be important to understanding what was available to adolescent girls in this setting and help to understand consumption. While we did not identify easily modifiable SDN in this setting, and adolescent girls' diets are unlikely to improve until poverty is addressed, further assessment of SDN in relation to the diet of adolescent girls in other LMIC settings is warranted. From our univariate analyses, researchers could consider prioritizing the assessment of education, occupation, food security, mental health, decision‐making autonomy and shared meals with family.

Given that dietary intake was poor in general, poverty alleviation strategies could play an important role. The government identifies the nutrition of adolescent girls as a priority for achieving the Sustainable Development Goals and recently outlined its adolescent nutrition strategy (Government of Pakistan, 2020b). This includes emphasis on the importance of counselling adolescent girls about diet and supplementation with iron and folic acid or multiple micronutrient supplements. Notably, while the evidence has shown that there is a role for micronutrient supplementation among adolescent girls, platforms by which to deliver the intervention have been less explored (Keats et al., 2021). We anticipate that the larger MaPPS Trial will help to inform whether multiple micronutrient supplementations affect adolescent girls' micronutrient status. There could also be a role for food fortification strategies, as micronutrient deficiencies are widespread nationally (UNICEF Pakistan, 2018).

Overall, within this population of adolescent girls living in rural Pakistan, we found that wealth quintile was more strongly associated with DDS than all other SDN investigated. As poverty has been identified as a barrier to dietary variability within multiple LMIC settings, this highlights the need to intervene at the root causes and value of implementing interventions that aim to reduce poverty, in addition to a multipronged approach at improving micronutrient intake.

## CONFLICT OF INTEREST

The authors declare they have no conflicts of interest.

## CONTRIBUTIONS

JBB and ZAB conceptualized the research question for this analysis. ZAB conceived the MaPPS Trial and secured funding. ZAB, JBB, YW, and SBS contributed to the design and writing of the MaPPS Trial protocol and data collection methods. YW and SBS implemented the study. YW oversees field operations, and IA oversees data management. MI and SC assisted JBB with the development of the data analysis plan and modelling method. DWS provided feedback on the research question, hierarchical design, framing, and interpretations. JBB produced the first manuscript draft. All authors reviewed the manuscript and approved the final version. ZAB is the guarantor.

## Supporting information


**Table S1.** Examining the differences between dietary recalls administered to participants in the MaPPS Trial dietary assessment subgroup
Figure S1. Reported consumption of the 10 food groups, disaggregated by wealth quintile, from participants' cumulative intake across the recalls (n = 390)

## Data Availability

Deidentified individual participant data that underlie the results reported in this article, code book, analytic code and protocol will be made available upon reasonable request by researchers who provide a methodologically sound proposal, pending application and approval, to achieve aims in the approved proposal following the publication of the article upon request to the corresponding author.
