# Supplementary material for: Dietary diversity and social determinants of nutrition among late adolescent girls in rural Pakistan
Source: Matern Child Nutr. 2021 Aug 31;18(1):e13265. doi: 10.1111/mcn.13265 (PMC8710090; doi:10.1111/mcn.13265)
Supplement: Supplementary file 1 — Table S1. Examining the differences between dietary recalls administered to participants in the MaPPS Trial dietary assessment subgroup Figure S1. Reported consumption of the 10 food groups, disaggregated by wealth quintile, from participants' cumulative intake across the recalls (n = 390) [file MCN-18-e13265-s001.docx]

**Supplemental Material**

**Conceptual framework**

To identify potential social determinants of nutrition, we reviewed the UNICEF framework on the causes of child malnutrition (UNICEF, 1998) and the WHO-developed framework on social determinants of health related to health and well-being (Commission on Social Determinants of Health, 2008). We then developed a conceptual framework that was intended to serve as a guide as to how underlying risk factors can affect nutritional status and downstream consequences among adolescent girls in Pakistan (Baxter et al., 2021). The ‘underlying causes’ level from the UNICEF framework was further split into two levels consistent with the WHO framework, depicting ‘structural’ and ‘intermediate’ determinants. The structural determinants included socioeconomic status, education, ethnicity, and occupation; the intermediate determinants included food security, hygiene and sanitation, health-related factors, mental well-being, food practices, and empowerment. Together, factors influenced the dietary intake of adolescent girls.

**Dietary diversity score (DDS) and minimum dietary diversity (MDD)**

DDS is an integer from 1–10 that serves as a measure of the adequacy of micronutrients within the diet, and derived from reported consumption of 10 standard, mutually-exclusive food groups in the past 24 hours (FAO & FHI 360, 2016). These groups include grains, white roots, and tubers (also called starchy staples); pulses (beans, peas, and lentils); nuts and seeds; dairy; meat, poultry, and fish (also called flesh foods); eggs; dark green leafy vegetables (e.g., spinach, mustard leaves); other vitamin A-rich fruits and vegetables (e.g., ripe mango, carrot, pumpkin); other vegetables (e.g., tomato, green pepper, onion); and other fruits (e.g., banana, apple). Reported food consumption data from the three 24-hour recalls for each participant were used to categorize consumed foods into the 10 food groups, which were summed to generate the DDS. Information on participants’ specific macro- and micronutrient intake from the 24-hour recalls will be published elsewhere. Participants’ DDS was used to generate MDD, which is a dichotomous proxy indicator for micronutrient adequacy based on whether at least five of the ten food groups have been consumed (FAO & FHI 360, 2016). MDD has been validated for 11 micronutrients.

**Food source assessment**

At each food recall, participants were also asked to identify the source of foods consumed over the past week by food group. Eight possible food sources were considered: own production; purchase; borrow/credit; barter/trade; work for food; gift; food aid; other. The food groups included the 10 standard food groups within the DDS tool, as well the 4 optional food groups, for a total of 14 food groups: foods made from grains; white roots and tubers; pulses (beans, peas, and lentils); nuts and seeds; milk and milk products; organ meat; meat and poultry; fish and seafood; eggs; dark green leafy vegetables; vitamin A rich vegetables, roots, and tubers; vitamin A rich fruits; other vegetables; other fruits.

**Cumulative DDS and MDD**

A cumulative DDS score was generated by summating each participant’s food group consumption across observations. As such, a food group was scored as 1 if a participant reported eating the food group at least once across the three 24-hour recalls. A cumulative MDD was also generated by applying a cut-off of ≥5 food groups to participants’ cumulative DDS to generate a binary variable.

**Explanatory variables**

Variables related to demographic information, socioeconomic status, and reproductive health were adapted from the Pakistan Demographic Health Survey tool (NIPS & ICF, 2019). A principal component analysis was applied to generate wealth quintile at the level of household, based on the participants’ home characteristics and reported household asset ownership (NIPS & ICF, 2019). Food security was assessed at the household-level, using the Household Food Insecurity Access Scale (Coates, Swindale, & Bilinsky, 2007). Self-reported health status, body image, and characteristics related to participants’ food consumption (i.e., skipping breakfast and sharing dinner with family) were adapted from the survey tool developed by the Health Behaviour in School-aged Children Study (Currie et al., 2014). Factors related to mental health (i.e., reported experience of depression, anxiety, and stress-related emotions in the previous week) were determined using the Depression, Anxiety, and Stress Scale 21-item assessment tool and applying set cut-offs to categorize participants scores as severe or extremely severe, moderate, mild, or none (Lovibond & Lovibond, 1995). Empowerment outcomes included generalized self-efficacy and decision-making autonomy. Self-efficacy scores were categorized into tertiles (i.e., low, moderate, and high) (Schwarzer & Jerusalem, 1995). Reported participation in 5 decisions (own food consumption, own health care, household food purchases, household food distribution, household purchases; adapted from Pakistan Demographic Health Survey) was used to generate a variable for decision-making autonomy (NIPS & ICF, 2019). In order to categorize participants’ involvement in decision-making into 3-categores (i.e., all decisions made by family, most decisions made by family, decisions made jointly with family or autonomously), a scoring system consistent with the Survey-based Women's emPowERment (SWPER) index for decision-making among women of reproductive age was used (Ewerling et al., 2017).

**Anthropometric nutritional status**

All anthropometric measures were collected in duplicate by two data collectors. There were pre-set allowable differences for measures (height: <1.0 cm; weight: <0.5 kg; MUAC: <0.5 cm), and in the case that two measurements did not meet the respective difference, a third measurement was taken. The average of two acceptable measures have been presented. Because of the overlap in age ranges between late adolescent girls (15 to 19 years) and women of reproductive age (WRA; 15 to 49 years), we also generated BMI and applied the suggested anthropometric cut-offs for WRA (i.e., short stature [attained height <145 cm] and BMI categories [underweight: <18.5 kg/m^2^; normal: 18.5-24.9 kg/m^2^; overweight: 25-29.9 kg/m^2^; obese: ≥30 kg/m^2^]) (Garcia & Mason, 1992; World Health Organization, 1995). The WHO Growth Reference typically is used to classify the growth of adolescents <19 years, thus the WHO igrowup package for Stata (StataCorp, College Station, TX) was applied to participants’ anthropometric measures to determine height-for-age and BMI-for-age z-scores (HAZ and BAZ, respectively) (de Onis et al., 2007). The corresponding WHO adolescent growth cut-offs were applied to generate estimates of stunting (HAZ <-2 SD) and categorize BMI (thinness: BAZ <-2SD; normal: -1 to 1 SD; overweight: >1 to 2 SD; obese: >2 SD).

**Micronutrient status**

All venous blood samples were collected in the field at participants’ homes. Two drops of whole blood were taken immediately to determine hemoglobin concentration using the HemoCue® Hb 301 System (HemoCue; Ängelholm, Sweden). The remaining sample was maintained at 2-8°C and transported to the field office for further processing. The serum was separated from the red blood cells following centrifugation and transferred to cryovials, which were stored at -80°C until analysis at the Nutrition Research Lab at the Aga Khan University (Karachi).

Micronutrient deficiencies of interest included anemia (determined from hemoglobin concentration), iron deficiency (ferritin concentration), vitamin A deficiency (retinol concentration), vitamin D deficiency (25[OH]D concentration) and inflammation (C-reactive protein [CRP] concentration). Ferritin and CRP were assessed using the immunoturbidimetric assay method (Cobas C311 Analyzer, Roche Diagnostics; ferritin kit: FERR4 [Tina‑quant Ferritin Gen.4, #04885317 190]; CRP kit: CRPLX [C-Reactive Protein (Latex), #20764930 322]); vitamin A using quantitative-high performance liquid chromatography photodiode array detection (Agilent HPLC, 1200/1260 Infinity Series with UV/PDA detection; manual method); and vitamin D using electrochemiluminescence protein binding assay (Diasorin Analyzer, LIAISON; kit: LIAISON® 25 OH Vitamin D TOTAL Assay, #310600).

Standard cut-offs were applied to determine the prevalence of the micronutrient deficiencies of interest. Anemia was defined as hemoglobin concentration <12 g/dL (World Health Organization, 2011a). Iron deficiency was defined per the WHO age and sex-specific specifications as a ferritin concentration of <15 ng/mL in the absence of inflammation, or an adjusted ferritin concentration of <70.0 ng/mL in the presence of inflammation, measured via CRP as a concentration of >5.0 g/L (World Health Organization, 2020). Iron deficiency anemia was determined from concurrent anemia and iron deficiency (World Health Organization, 2017). To determine vitamin A deficiency, the general WHO cut-off for populations of retinol concentration <0.7 µmol/L was applied (World Health Organization, 2011b). Vitamin D deficiency was defined as 25(OH)D concentration <20 ng/mL (Institute of Medicine, 2011)

**Table S1. Examining the differences between dietary recalls administered to participants in the MaPPS Trial dietary assessment subgroup**

| **Comparison** | **Difference**  (mean ± SD [range]) | **Histogram** |
| --- | --- | --- |
| DDS between recalls 1 and 2  (n = 390) | -0.10 ± 1.15 [-3 to 3] |  |
| DDS between recalls 1 and 3  (n = 390) | -0.17 ± 1.20 [-4 to 4] |  |
| DDS between recalls 2 and 3  (n = 390) | -0.06 ± 1.18 [-3 to 4] |  |

Abbreviation: DDS, dietary diversity score


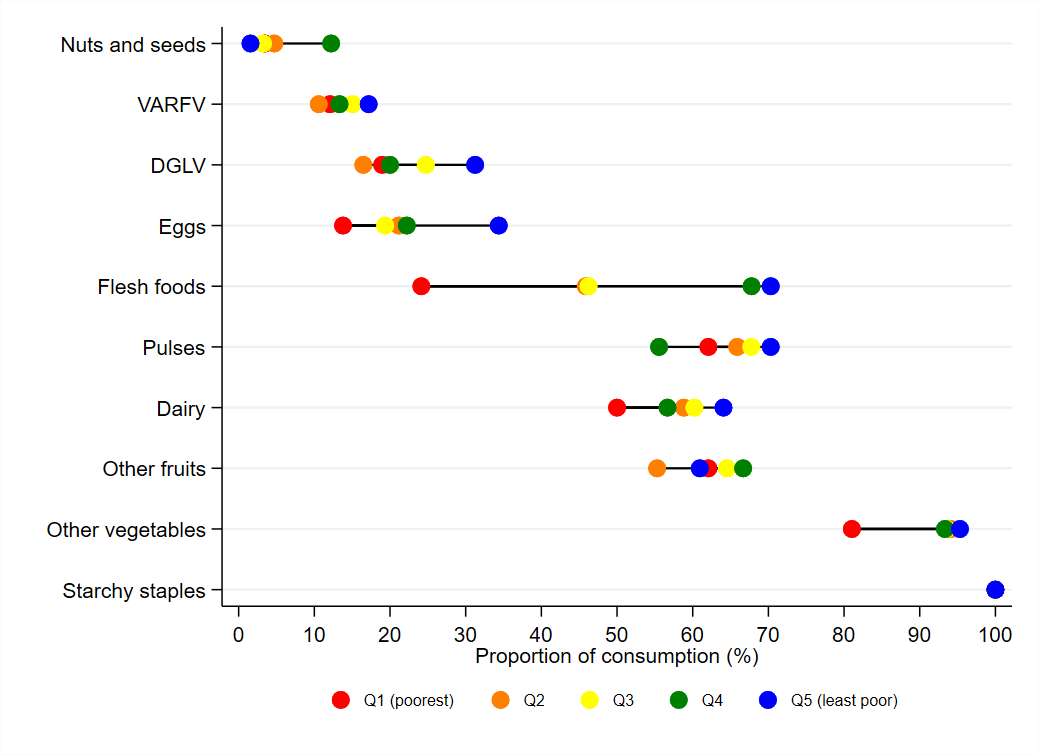


**Figure S1. Reported consumption of the 10 food groups, disaggregated by wealth quintile, from participants’ cumulative intake across the recalls (n = 390)**

**References**

Baxter, J. B., Wasan, Y., Hussain, A., Soofi, S. B., Ahmed, I., & Bhutta, Z. A. (2021). Characterizing Micronutrient Status and Risk Factors among Late Adolescent and Young Women in Rural Pakistan: A Cross-Sectional Assessment of the MaPPS Trial. *Nutrients, 13*, 1237.

Coates, J., Swindale, A., & Bilinsky, P. (2007). *Household food insecurity access scale (HFIAS) for measurement of household food access: Indicator guide (v. 3)*. Washington: Food And Nutrition Technical Assistance Project, Academy for Educational Development.

Commission on Social Determinants of Health. (2008). *Closing the gap in a generation: health equity through action on the social determinants of health*. Geneva: World Health Organization.

Currie, C., Inchley, J., Molcho, M., Lenzi, M., Veselska, Z., & Wild, F. (2014). *Health Behaviour in School-aged Children (HBSC) Study Protocol: Background, Methodology, and Mandatory items for the 2013/14 Survey*. St. Andrews: CAHRU.

de Onis, M., Onyango, A., Borghi, E., Siyam, A., Nishida, C., & Siekmann, J. (2007). Development of a WHO growth reference for school-aged children and adolescents. *Bull World Health Organ, 85*, 660–667.

Ewerling, F., Lynch, J. W., Victora, C. G., van Eerdewijk, A., Tyszler, M., & Barros, A. J. D. (2017). The SWPER index for women's empowerment in Africa: development and validation of an index based on survey data. *Lancet Glob Health, 5*(9), e916-e923. doi:10.1016/s2214-109x(17)30292-9

FAO, & FHI 360. (2016). *Minimum Dietary Diversity for Women: A Guide for Measurement*. Rome: Food and Agricultura Organization of the United Nations.

Garcia, M., & Mason, J. (1992). *Second Report on the World Nutrition Situation – Volume I: Global and Regional Results. A Report compiled From Information Available to the United Nations Agencies of the ACC/SCN*. Geneva: World Health Organization.

Institute of Medicine. (2011). *Dietary Reference Intakes for Calcium and Vitamin D*. Washington (DC): National Academies Press (US).

Lovibond, S. H., & Lovibond, P. F. (1995). *Manual for the Depression Anxiety Stress Scales*. Sydney: Psychology Foundation.

NIPS, & ICF. (2019). *Pakistan Demographic and Health Survey 2017-18*. Islamabad, Pakistan, and Rockville, Maryland, USA: NIPS and ICF.

Schwarzer, R., & Jerusalem, M. (1995). Generalized self-efficacy scale. In J. Weinman, S. Wright, & M. Johnston (Eds.), *Measures in health psychology: a user’s portfolio. Causal and control beliefs* (pp. 35–37). Windsor: NFER-NELSON.

UNICEF. (1998). *The State of the World’s Children [Internet]*. New York: Oxford University Press.

World Health Organization. (1995). *Physical status: the use and interpretation of anthropometry*. Geneva: World Health Organization.

World Health Organization. (2011a). *Haemoglobin concentrations for the diagnosis of anaemia and assessment of severity. Vitamin and Mineral Nutrition Information System*. Geneva: World Health Organization.

World Health Organization. (2011b). *Serum retinol concentrations for determining the prevalence of vitamin A deficiency in populations. Vitamin and Mineral Nutrition Information System*. Geneva: World Health Organization.

World Health Organization. (2017). *Nutritional anaemias: tools for effective prevention and control*. Geneva: World Health Organization.

World Health Organization. (2020). *WHO guideline on use of ferritin concentrations to assess iron status in individuals and populations*. Geneva: World Health Organization.
